# Noninvasive Biomarkers of Colorectal Cancer: Role in Diagnosis and Personalised Treatment Perspectives

**DOI:** 10.1155/2018/2397863

**Published:** 2018-06-13

**Authors:** Gianluca Pellino, Gaetano Gallo, Pierlorenzo Pallante, Raffaella Capasso, Alfonso De Stefano, Isacco Maretto, Umberto Malapelle, Shengyang Qiu, Stella Nikolaou, Andrea Barina, Giuseppe Clerico, Alfonso Reginelli, Antonio Giuliani, Guido Sciaudone, Christos Kontovounisios, Luca Brunese, Mario Trompetto, Francesco Selvaggi

**Affiliations:** ^1^Unit of General Surgery, Department of Medical, Surgical, Neurological, Metabolic and Ageing Sciences, Università degli Studi della Campania “Luigi Vanvitelli”, Piazza Miraglia 2, 80138 Naples, Italy; ^2^Colorectal Surgery Unit, Hospital Universitario y Politécnico La Fe, Valencia, Spain; ^3^Department of Medical and Surgical Sciences, OU of General Surgery, University of Catanzaro, Catanzaro, Italy; ^4^Department of Colorectal Surgery, Clinic S. Rita, Vercelli, Italy; ^5^Institute of Experimental Endocrinology and Oncology (IEOS), National Research Council (CNR), Via S. Pansini 5, Naples, Italy; ^6^Department of Medicine and Health Sciences, University of Molise, Via Francesco de Sanctis 1, 86100 Campobasso, Italy; ^7^Department of Abdominal Oncology, Division of Abdominal Medical Oncology, Istituto Nazionale per lo Studio e la Cura dei Tumori, “Fondazione G. Pascale, ” IRCCS, Naples, Italy; ^8^1st Surgical Clinic, Department of Surgical, Oncological, and Gastroenterological Sciences, University of Padua, Padua, Italy; ^9^Dipartimento di Sanità Pubblica, Università degli Studi di Napoli Federico II, Naples, Italy; ^10^Department of Colorectal Surgery, Royal Marsden Hospital, London, UK; ^11^Department of Internal and Experimental Medicine, Magrassi-Lanzara, Institute of Radiology, Università degli Studi della Campania “Luigi Vanvitelli”, Piazza Miraglia 2, 80138 Naples, Italy; ^12^Department of Medicine and Health Sciences “V. Tiberio”, University of Molise, Campobasso, Italy; ^13^Department of Surgery and Cancer, Chelsea and Westminster Hospital Campus, Imperial College London, London, UK

## Abstract

Colorectal cancer (CRC) is the third leading cause of cancer-related deaths worldwide. It has been estimated that more than one-third of patients are diagnosed when CRC has already spread to the lymph nodes. One out of five patients is diagnosed with metastatic CRC. The stage of diagnosis influences treatment outcome and survival. Notwithstanding the recent advances in multidisciplinary management and treatment of CRC, patients are still reluctant to undergo screening tests because of the associated invasiveness and discomfort (e.g., colonoscopy with biopsies). Moreover, the serological markers currently used for diagnosis are not reliable and, even if they were useful to detect disease recurrence after treatment, they are not always detected in patients with CRC (e.g., CEA). Recently, translational research in CRC has produced a wide spectrum of potential biomarkers that could be useful for diagnosis, treatment, and follow-up of these patients. The aim of this review is to provide an overview of the newer noninvasive or minimally invasive biomarkers of CRC. Here, we discuss imaging and biomolecular diagnostics ranging from their potential usefulness to obtain early and less-invasive diagnosis to their potential implementation in the development of a bespoke treatment of CRC.

## 1. Introduction

Colorectal cancer (CRC) is the third most common cancer among men and women and the third leading cause of cancer-related deaths in the world, with an incidence of 1.2 million new cases and 608,700 deaths annually [1].

Metastasis accounts for approximately 90% of CRC-related deaths; this is mainly due to the absence of an ideal method of screening [2]. Detection of CRC at an early stage may confer a 90% 5-year survival rate, compared to 12% if distant metastasis occurs [3, 4].

One of the primary targets of screening is the identification of advanced colorectal adenomas.

The currently available screening modalities, such as the guaiac-based faecal occult blood test (gFOBT) and carcinoembryonic antigen (CEA) test, are effective but limited by low specificity and sensitivity. Sigmoidoscopy and colonoscopy are invasive, have certain morbidity risks, and require cumbersome preparatory procedures that lead to a low participation rate.

The gFOBT has been associated with a reduction of 15–33% in CRC-related mortality, particularly if the test is performed every 1 or 2 years [5, 6]. Despite being noninvasive, inexpensive, and easily applicable, it has low accuracy, particularly regarding the detection of preneoplastic lesions; it also has a low specificity rate leading to a high number of unnecessary colonoscopies [7, 8]. The new, more sensitive version of an antibody-based globin test, known as immunochemical FOBT or faecal immunochemical test (FIT), is inconvenient because the specimen needs to be sent to a laboratory for testing [9]. Nowadays, colonoscopy is the gold standard for the early diagnosis of CRC [10], but it has several risks such as bleeding, perforation, missed adenoma/cancer, and related death.

The ideal CRC biomarker should be easily and quantitatively measured, highly specific, and sensitive, as well as reliable and reproducible [11]. It should be able to stratify between different risk-based populations, selecting patients who really need a second-line test (endoscopic and radiologic investigations). Ideally, this aim can be achieved with a noninvasive and inexpensive method, using easily available biological samples such as urine, breath, serum, and faeces.

Despite the advances made over the last years, no single test is currently able to diagnose and monitor the posttreatment course of CRC patients. Herein, we review the current status of noninvasive biomarkers in CRC and provide insights for their implementation in the clinical management of patients.

## 2. Circulating Biomarkers and Eliminated Metabolites

### 2.1. Genetic and Epigenetic Alterations and CRC

Genetic and epigenetic changes characterizing the carcinogenesis of CRC are essential for the identification and development of an ideal biomarker [12]. Genetic markers are based on the identification of mutations in a subset of genes, including p53, APC, KRAS, NRAS, and DNA repair genes such as hMSH1 (human mutS homolog 1) or hMLSH2 [13, 14]. Unfortunately, this approach has a modest diagnostic sensitivity for invasive cancers and advanced benign tumors [15]. Epigenetic alterations include DNA methylation, microRNA (miRNA) expression, histone modification, and chromatin remodelling. They represent inheritable changes in gene expression without modifications to the DNA sequence.

DNA methylation consists in the enzymatic addition of a methyl group to cytosine in 5-position. The process is catalyzed by DNA methyltransferases and usually entails a covalent linkage within a CG dinucleotide sequence, termed CpG transcription [16].

Owing to their high tissue specificity and critical role in oncogenesis, miRNAs have the potential to be reliable biomarkers for the diagnosis and classification of CRC as well as for predicting treatment outcomes in the near future [17–19]. Several studies have recently demonstrated the role of miRNAs obtained from different body fluids (such as plasma, serum, urine, saliva, and tissues) in the pathogenesis of CRC (including metastasis spread [20]) with subsequent implications on treatment and prognosis [21].

The improvement of validated protocols and the discovery of new technologies such as next-generation sequencing (NGS) [22] allow a very careful evaluation of the whole miRNAome in different samples.

miRNAs are small single-stranded, noncoding RNAs, discovered in 1993 as developmental regulators in *Caenorhabditis elegans* [23, 24], with a length of 18–25 nucleotides [25]. Their aberrant expression patterns have been detected in various types of malignancies including breast cancer, lung cancer, pancreatic cancer, ovarian cancer, and CRC [26–28], playing an essential role as posttranscriptional regulators of carcinogenesis, progression, invasion, angiogenesis, and metastasis [25, 29–34].

They suppress translation or induce mRNA degradation by binding to the 3′ untranslated region (UTR) of their target genes [35]. More than 50% of the discovered human miRNA genes are localized in fragile chromosomal regions that are susceptible to amplification, deletion, or translocation during the natural history of CRC [36, 37]. This makes them the most promising future predictive markers for the diagnosis and prognosis of CRC; additionally, they could aid to determine the therapeutic response to chemotherapeutic drugs.

After several enzymatic reactions, the mature miRNA is integrated into the RNA-induced silencing complex (RISC) to then negatively regulate the expression of hundreds of target mRNAs by translation inhibition or mRNA degradation. This is achieved by the recognition of complementary sites on the target mRNA [38]. Consequently, miRNAs are able to give us more prognostic and diagnostic information than mRNAs. miRNAs also modulate T and B lymphocyte activation (both the innate and adaptive immune responses [39]), thus helping cancer cells avoid recognition by the immune system in the blood/lymph vessels.

Lastly, they target inflammatory signalling molecules, thereby inducing or inhibiting chronic inflammation and inflammation-related cancers. This is confirmed by studies on the expression of KRAS, which is inversely correlated with miR 143 [40] and c-Myc, which can promote tumoral growth via miR 17-92 [41]. Both miR 324-5p and miR 122 are involved in the regulation of TNF-alfa [42], CUEDC2 [43], and NOD2 [44].

#### 2.1.1. Plasmatic miRNAs

Several miRNAs are dysregulated in the plasma of patients with CRC [45, 46]. They can either circulate freely or be in exosomes. Thanks to their small size, miRNAs are well protected from endogenous degradation [47–49] and can remain stable for a long period of time, in contrast to the fast degradation of mRNAs and proteins. Furthermore, cancer cells secrete some miRNAs into systemic circulation [48], confirming their central role in CRC screening.

The number of aberrantly expressed miRNAs in CRC tissues has rapidly grown due to the increasing number of studies on the topic [28, 50]. As an example, miR 17-92a has an oncogenic function because it is upregulated during the well-known adenoma-to-carcinoma sequence [51, 52].

miR 21, one of the most extensively investigated oncogenic miRNAs, is highly correlated with CRC cell proliferation, invasion, lymph node metastasis, and advanced clinical stage [53–55]. It is overexpressed in colorectal adenomas when compared with normal colonic mucosa [54]. It participates in the multistep process of CRC carcinogenesis, regulates several pathways such as MAPK and WNT/Beta-catenin [56–58], and its level decreases after surgical removal of CRC [59].

Other evaluated miRNAs are linked with hepatic metastasis. These can also be useful for early detection of CRC, as predictors of recurrence of CRC (stages II and III), and to determine the probability of resistance to preoperative chemoradiotherapy (CRT) in a CRC cell line [46, 60–67]. miR 92a is overexpressed in serum, plasma, and stool of patients with advanced adenoma, when compared to controls [66–68].

#### 2.1.2. Faecal miRNAs

Exfoliated faecal colonocytes or tumor-secreted miRNAs are directly and continuously released from tumors into the intestinal lumen, providing a rationale for a stool-based miRNA test for the diagnosis of CRC [11, 69]. Furthermore, miRNAs in faeces correlate with the grading of the tumor [70]. Faecal miR 135b is elevated in CRC and adenomatous tissue samples in contrast to adjacent healthy tissue [71], whereas miR 106a can decrease the number of false negatives when using a gFOBT [72]. Unfortunately, the stool environment is much more complex (compared to plasma) and its testing requires a certain volume and density of the sample for each assay.

#### 2.1.3. miRNAs, Diet, and Lifestyle

In CRC, there is a clear link between lifestyle, diet, and epigenetic factors expressed in an aberrant way. However, it is still debated whether changes in lifestyle can modify epigenetic mechanisms and reduce the risk of CRC progression.

Tarallo et al. [73] demonstrated the modulatory effect of different dietary habits on a panel of miRNAs; the highest differences in the expression (in stool and plasma samples) of miR 17-92 cluster among people with vegan, vegetarian, or omnivorous diet habits.

Several recent studies show that fish; oil-fed animals; vitamins A, D, and E; and minerals such as selenium and resveratrol (trans-3,4′,5′-trihydroxystilbene) can modify the levels of expressed miRNAs [73–79].

Various recent studies demonstrate a clear relationship between miRNA expression and CRC [80]. These new diagnostic possibilities are highly influencing the current research in the CRC field.

Endogenous miRNAs, packed and protected from the action of RNase (in contrast with rapid degradation of mRNA and proteins), allow us to discriminate normal colonic mucosa, colon adenomas, and carcinomas. The possibility of miRNA-based therapies, inhibiting oncogenic miRNAs or restoring tumor suppressor miRNAs, could open a new scenario in the treatment of CRC, despite the bias on the different methods for evaluating the population, methodology of collection of the used samples, and quantification methods.

#### 2.1.4. Methylated DNA

Increased concentrations of circulating methylated DNA have been reported in the blood of cancer patients [81–83]. *SEPT9* is one of the most widely studied genes with an important role in the early diagnosis of CRC as well as in metastatic CRC.

In the PRESEPT study, Church and coworkers [84] found 666 (9.7%) advanced and 2359 (34.3%) nonadvanced adenomas in the 6874 patients who underwent colonoscopy. Among them, circulating methylated *SEPT9* has been identified in 9.6% of the advanced adenomas and 7.7% of the nonadvanced adenomas.

Barault et al. [85] identified candidate biomarkers of CRC analysing the methylation profile of CRC cell lines. Methylated ctDNA enables, in association with CT scan, the tracking of tumor response in metastatic CRC patients treated with chemotherapy (FOLFOX, FOLFIRI, ± bevacizumab) and targeted agents (panitumumab). They validated its use in monitoring metastatic CRC response to therapy, including chemotherapy, targeted therapy, and temozolomide in a longitudinal study. Furthermore, several authors evaluated the diagnostic performance of SEPT9 assay along with other blood-based methylated genes. The association of SEPT9 with TAC1 methylation assay yielded a sensitivity of 73.1% and a specificity of 92.3% [86] while its association with *TMEFF2* and *ALX4* further increased both sensitivity (80.7%) and specificity (90.0%) [87].

### 2.2. Neurotensins and CRC

There is increasing recognition that cancers of the gastrointestinal tract (pancreas as well as other organs) express receptors for various endogenous host hormones. This raises the possibility that hormones can play a role in the proliferation of these cancers and therefore highlights the potential of these hormonal signalling pathways as targets for novel cancer diagnostic and therapeutic strategies. One of these promising candidates in CRC is the tridecapeptide neurotensin (NT) [88, 89]. NT was first isolated in 1973 from the bovine hypothalamus and digestive tract [90]. Its physiological functions are those of a neurotransmitter in the central nervous system and of a hormone in the periphery. There is increasing evidence of the role it plays in CRC.

Some colonic tumors synthesise and release NT, resulting in autocrine control and cellular proliferation [91]. Physiological levels of NT appear to stimulate the growth of many human CRC cell lines (SW480, SW620, HT29, HCT116, and Cl.19A) expressing the NT receptor 1 (NTR1) [92]. NT accelerates colonic cancer carcinogenesis in animal models. For example, rats injected with both a CRC carcinogen and NT demonstrate a significant increase in the number, size, and invasiveness of colon tumors [93]. Administration of NT by itself significantly stimulated growth in murine colon tumors as well as human colon cancers xenografted into mice; it also resulted in a significant decrease in survival [94].

In humans, NT mRNA, peptide, and receptor were found in resected CRC specimens as well as four well-known human cancer cell lines *in vitro*. In surgical specimens where NT was identified in cancer cells, it was absent in adjacent normal bowel mucosa [91].

Gui et al. examined NTR1 expression in human CRC by measuring NTR1 mRNA in normal colonic mucosa, adenomas, and colonic adenocarcinomas. NTR1 mRNA expression was undetectable in epithelial cells of normal colonic epithelium, but it was expressed in adenomas and adenocarcinomas. Higher expression levels were seen in adenocarcinomas when compared to adenomas. Tissue from lymphovascular invasion showed even higher expression levels of NTR1 than that from the rest of the tumor. These results suggest that increased NTR1 expression may be an early event during colonic tumorigenesis that can also contribute to tumor progression and aggressive behaviour in colonic adenocarcinomas [95].

Evaluation of blood NT levels in colorectal cancer was recently conducted using 56 colorectal cancer patients and 15 controls; early evidence suggests that NT levels could differentiate between cancer and noncancer patient groups.

### 2.3. Liquid Biopsy

Biopsies have a central role in disease management, particularly in cancer patients. They allow clinicians to diagnose, determine a treatment course, and evaluate prognosis. In addition to specifying the histological nature of the disease, tissue biopsies are used to determine the genetic features of the tumor. This information can be used to treat patients with drugs tailored to the genetic makeup of their tumor and to give predictive and prognostic information.

However, although tissue biopsies are critical in the decision-making process, they have limitations. A single biopsy represents a snapshot of the complexity of molecular tumor alterations and tends to underestimate the real intratumoral heterogeneity [96]. Moreover, molecular targeted therapies may require multiple biopsies to accurately evaluate both the intratumoral heterogeneity and the genome modifications occurring during treatment because cancer genomes are also unstable and tend to change over time. Obtaining multiple biopsies at baseline and during the treatments is challenging owing to patient discomfort, procedural complications, costs, tumor accessibility, and the potential risk of tumor seeding.

In the field of precision medicine, the term liquid biopsy (LB) refers to those genetic tests performed on a biological component extracted from body fluids, in particular, from whole blood. This sample can be used to obtain circulating tumor cells (CTC), circulating tumor DNA (ctDNA), and exosomes [97, 98]; these components represent a small fraction of the total biological elements actively or passively released into the blood through metastisation processes, necrosis, or apoptosis. Today, many clinical trials have aimed to investigate the role of LBs in the management of metastatic CRC (mCRC) patients, specifically by analysing the role of CTC, ctDNA, and exosomes as alternative biological sources to monitor tumor evolution and response in a dynamic manner, considering that cancer is not a “molecularly stable” disease [99–101]. In addition, an LB represents a key approach to analyse, through a noninvasive and simple blood test, the molecular heterogeneity among different tumor sites in the same patient (primary tumor versus distant metastasis) to define the best target for a therapeutic approach or to monitor patients with no clinically detectable disease after surgery and standard therapy [99–106].

Plasma DNA that is analysed on CTC or on ctDNA has been suggested as an alternative way to evaluate tumor genomes [106–112]. CTCs are cancer cells derived from tumors that are released into the bloodstream through a process known as the epithelial-mesenchymal transition (EMT), from either a primary or metastatic site. In the past years, the simple presence of CTCs was an indicator of a poorer prognosis in CRC and other cancer types, such as breast, prostate, and lung cancer. CTC molecular characterization represents an attractive real-time option to monitor metastatic diffusion before instrumental detection [98–100].

Plasma cell-free DNA (cfDNA) is the result of DNA fragments that are released into circulation from both normal and tumor cells. Since CTC and ctDNA are valid sources to evaluate tumor genomes and can be considered as “surrogates” of tissue biopsies, they can be defined as LB. Initially, both CTC and ctDNA were used as simple quantitative markers. The analysis of clinical relevant mutations can also be feasible in a simple way by using ctDNA, even if it represents a small fraction of the total cfDNA released into the blood by healthy cells or primary cancer cells or directly by CTCs. Additionally, the quantity of cfDNA has a prognostic role in mCRC patients, and, overall, cfDNA levels have been demonstrated to be higher in cancer patients, when compared to healthy controls [101, 103, 105, 106].

In another recent study, Strickler et al. [113] reported results from clinical cfDNA testing of 1397 patients with advanced CRC. They compared these results with three large CRC tumor tissue sequencing cohorts. Evidence of ctDNA in the blood was similar to recent studies in colon cancer. Furthermore, authors identified a previously unreported cluster of EGFR extracellular domain (ECD) mutations involving V441 and S442 that accounted for 25% of all ECD mutations, representing an important and novel mechanism of resistance to EGFR blockade.

Analysis of plasma samples may offer several advantages in determining KRAS mutation status in patients who have progressed on EFGR target therapy. Siena et al. [114] studied the mechanism of secondary resistance in EGFR inhibitor-treated patients finding new *KRAS* mutations in 7.1% and 57.1% of patients whose tumor genotype was determined using tissue­based and plasma­based analyses, respectively, during treatment with the combination of irinotecan and panitumumab.

Exosomes are very interesting, small endocytic membrane vesicles that are initially isolated from the peripheral circulation of cancer patients and play a central role in the communication processes among cells by activation of surface ligands or by transferring of molecules among the cells. Exosomes can either manipulate the local and systemic environment, allowing cancer growth and dissemination, or modulate the immune system to elicit or suppress an antitumor response. In addition, exosomes represent a good source of DNA fragments, proteins, mRNA, miRNA, and other biological molecules and are protected by a lipid bilayer membrane, which confers a high degree of stability. Recently, many studies on exosomes demonstrate either a prognostic or predictive value, emphasizing their potentiality in clinical practice. A genome-wide expression profile of miRNAs has been shown to be significantly different among primary lung cancers and corresponding noncancerous lung tissues and thus has shown to have a potential role as a diagnostic marker [98, 105].

Therefore, in the near future, an integrated analysis of ctDNA, CTC, and exosomes could be used in a clinical setting for mCRC to refine patient therapy selection and management.

Currently, there is an increasing interest in the evaluation of genomic features available from LB.

The concept behind LB and its possible clinical applications in CRC is summarized in Table 1.

#### 2.3.1. LB as a Diagnostic Biomarker

The first effort towards the clinical use of cfDNA from LB was the simple quantitative evaluation of plasma DNA. A significant difference in cfDNA levels was found in healthy subjects compared to cancer patients. Heitzer et al. [115] found that, compared to healthy subjects, stage IV CRC patients (*n* = 32) showed higher cfDNA levels with substantial variability. Moreover, a third of patients had a biphasic size distribution of plasma DNA fragments, and this finding was associated with increased CTC numbers and an elevated concentration of KRAS-mutated plasma DNA fragments. Therefore, CRC patients show not only higher levels of cfDNA but also a specific pattern of tumor DNA fragmentation.

Frattini et al. [116] performed a quantitative analysis of plasma DNA in 70 CRC patients and 20 healthy subjects at baseline and during follow-up. In a subset of CRC patients, they also compared the KRAS mutation and the p16INK4a promoter hypermethylation in tissue samples and in cfDNA. They found that plasma DNA levels are useful for diagnosing cancer as well as determining disease-free status and the presence of recurrence.

Unfortunately, the fraction of ctDNA originating from tumor cells is between 0.01% and more than 90%, and it can vary greatly [117].

#### 2.3.2. LB as a Prognostic Biomarker

Clinical, radiological, histopathological, and molecular factors are widely used as prognostic factors of rectal cancer. Tumor alterations in LB could have the potential to be associated with prognosis. Lecomte et al. [118] evaluated KRAS mutations and epigenetic alterations such as hypermethylation of a cyclin-dependent kinase inhibitor in cfDNA of 8 stage I, 21 stage II, 16 stage III, and 13 stage IV CRC patients. KRAS mutations and epigenetic alterations were found in 20 to 50% of these patients, and all of the patients without evidence of KRAS mutations or epigenetic alterations showed a 2-year survival rate. They also found an association between plasma ctDNA levels and the prognosis of CRC patients. These findings were confirmed by Diehl et al. [117], who demonstrated that CRC patients who relapsed within 1 year after surgery had higher ctDNA levels at the time of recurrence.

Bertorelle et al. [119] evaluated the association between RNA-hTERT (telomere-specific reverse transcriptase) plasma levels and the overall survival of stage II CRC patients, for whom the value of adjuvant chemotherapy is still debated. Compared to patients with low hTERT levels, those with high hTERT levels showed a significantly poorer survival rate (hazard ratio = 3.30, 95% CI 1.98–5.52), suggesting that hTERT levels could support the decision of performing adjuvant chemotherapy in stage II CRC patients.

In the CAPRI-GOIM trial, conducted by Normanno et al. [120], 340 KRAS exon-2 wild-type metastatic CRC patients received first-line cetuximab plus FOLFIRI. Tumor samples were analysed using NGS while BEAMing (digital PCR technology combines emulsion PCR with magnetic beads and flow cytometry for the highly sensitive detection and quantification of mutant tumor DNA molecules) has been used to search for KRAS and NRAS mutations in plasma samples. They concluded that ctDNA may replace tumor tissue analysis.

#### 2.3.3. LB as a Predictive Biomarker

The prediction of tumor responses to a neoadjuvant therapy is clinically relevant because it can allow for treatment modifications before or during the treatment and, ultimately, to a tailored therapy that avoids inefficient, toxic, and costly approaches. Moreover, a treatment fails when resistance develops against chemotherapeutic agents, as observed for KRAS in CRC patients [121]. In this setting, LB could be preferable to tissue biopsies to monitor molecular changes throughout therapy (e.g., biological drugs), thus avoiding repeated tumor tissue sampling; it could also be useful to detect drug resistance before it becomes clinically evident. Kuo et al. [122] compared KRAS mutations in cfDNA and primary tumor tissues and demonstrated that the detection rate of KRAS mutations was 50% in plasma and 28.8% in resected primary tumor tissue with an agreement of 78.8%. Diaz et al. [123] showed that, in CRC patients without KRAS mutations, treatment with panitumumab induced mutations in 38% of cases within 5 and 6 months following treatment. In a blind prospective study, Thierry et al. [109] compared KRAS and BRAF mutation statuses in tumor tissue and cfDNA of mCRC patients. They showed a 100% diagnostic specificity and sensitivity for the BRAF V600E mutation and a 98% specificity and 92% sensitivity for the KRAS mutation by cfDNA analysis. In 98 clinically stage II-III rectal patients who underwent neoadjuvant CRT, RNA-hTERT plasma levels were found to be a promising biomarker of tumor response [124]. The posttherapy levels of hTERT statistically decreased, and the difference of cfRNA levels between post- and preneoadjuvant therapy independently predicted tumor response. Agostini el al. [125] evaluated the role of cfDNA as a predictor of tumor response in rectal cancer patients who underwent neoadjuvant CRT. Based on the findings that cfDNA arising from tumor cells can be recognized on the basis of fragment lengths (compared to physiological cfDNA), they found that the longer fragments of cfDNA (derived from tumor cells) and, in particular, the ratio between long and short fragments (derived from apoptosis), were associated with tumor response to neoadjuvant therapy.

#### 2.3.4. LB as a Biomarker of Tumor Relapse

Another promising clinical application of an LB is the detection of tumor relapse after a curative treatment. Currently, local or distant recurrence is detected by clinical data and radiological imaging. These methods are costly with a questionable cost-effective value. An LB has the potential to overcome this limitation. Diehl et al. [117] demonstrated that it was possible to detect disease recurrence by monitoring tumor-specific alterations in the plasma of CRC patients after surgery with almost 100% sensitivity and specificity. The persistence of tumor alterations in cfDNA after a radical surgery was associated with an incomplete resection, thus allowing clinicians a very early identification of residual disease in patients. Frattini et al. [116] reported the role of cfDNA as a promising biomarker of recurrence; however, CEA determination is currently, even with its limitations, the only widely accepted biomarker used in clinical practice.

Resection radicality is one of the most important predictors for local recurrence and overall survival. In the largest prospective trial of minimal residual disease (MRD) to date, Tie et al. [126] performed next-generation-sequencing of 1046 plasma samples from 230 patients with resected stage II colorectal cancer. Thanks to the early decreases in ctDNA amounts in patients with metastatic disease, they demonstrated that plasma tumor DNA is a better marker for recurrence than carcinoembryonic antigen (CEA), which is currently used in the clinical setting.

The biological principles behind an LB are widely accepted, and the future applications are appealing. Although many studies support the role of LB as a new noninvasive tool in cancer detection and cancer-treatment settings, few studies have focused on the impact of using LB in the diagnosis and treatment of rectal cancer. Moreover, because this research topic is still relatively new, it is quite difficult to translate early findings into clinical applications. In addition, there are many technical aspects that differ between studies with a lack of standardization, which makes clinical application even more difficult. These considerations led to the conclusion that, although there is a solid theoretical basis and increasing evidence for its potential clinical use, the inclusion of LB into the clinical decision-making process for CRC diagnosis and treatment will require more time.

## 3. Imaging

The use of imaging in CRC has significantly evolved over the last decade, playing a key role in providing answers concerning diagnosis, staging, treatment optimization, and follow-up [126–131]. The imaging modalities currently available for CRC assessment (Table 2) can be divided into two main types: anatomical and functional. Anatomical imaging modalities still remain the mainstay, with computed tomography (CT) imaging suited for colon tumor evaluation and magnetic resonance imaging (MRI) optimal for rectal tumor assessment. However, with the development of new tracer and contrast agents, the evolution of fusion technologies between fludeoxyglucose positron emission tomography (FDG-PET) and MRI and the development of functional MRI techniques may offer new perspectives into cancer perfusion, metabolic, and molecular phenotypes [132]. During recent years, MRI has gained wide acceptance in the assessment of CRC and is considered the first-choice imaging modality for the primary staging and restaging after CRT [133–135]. In particular, despite CT, MRI is an imaging technique that provides functional data in addition to structural and anatomic details. Diffusion-weighted MRI (DW-MRI) and dynamic contrast-enhanced MRI (DCE-MRI) tools can allow to evaluate biological and functional modifications induced by treatment, also aiming to predict clinical outcomes in the setting of adjuvant therapies [136].

DW-MRI investigates and highlights the random movement (“diffusion”) of water protons in the extracellular space of biological tissues and derives its imaging contrast from these differences. The diffusion of water molecules in biological tissues depends on many factors and is mainly influenced by cellular density [134, 137]. In tissues with low cellularity, water molecules can freely diffuse resulting in a low DW-MRI signal. On the contrary, this mobility is impeded or “restricted” in tissues with high cellularity (e.g., a tumor) due to reduced extracellular space, resulting in a high DWI-MRI signal. Water proton diffusion can be quantified by the means of the apparent diffusion coefficient (ADC), which reflects the degree of restriction of water molecules (diffusion), indirectly reflecting tissue cellularity [134, 136].

Recognizing these properties, DW-MRI can be a useful tool to detect CRC in cases where the identification of cancer with conventional MRI sequences and CT may be difficult. This technique is useful in cases including malignant transformation within nonspecific mural thickening, desmoplastic reaction, fibrotic or inflammatory changes due to inflammatory bowel disease (IBD), pelvic extraintestinal malignancy, or radiotherapy [138–141]. The addition of DWI-MRI to the conventional T2-weighted sequences improves lesion conspicuity of rectal cancer with 96% sensitivity and a positive predictive value of up to 100% [142, 143]. In this regard, Barral et al. reported that DW-MRI is able to reveal malignant foci in rectal involvement by IBD [140]. Similarly, several studies suggested that DWI-MRI also increases the sensitivity for the diagnosis of colon cancer, with the ability to discriminate between colon cancer and acute diverticulitis in patients with uncertain CT findings (due to a pseudotumoral diverticulitis pattern [142–145]). The use of DWI-MRI could also aid to exceed the limitation of conventional MRI sequences in discriminating between T2 and T3 cancer because the former may present with a desmoplastic reaction resembling cancer invasion.

By quantitative analysis, the ADC value of rectal cancer is reported to be significantly lower than that of a normal rectal wall, with a threshold ADC value of 1.240 × 10–3 mm^2^/s having a sensitivity of 94% and a specificity of 100% for the diagnosis of rectal cancer [146]. The ADC value has been proposed as a potential biomarker for rectal cancer because it seems to correlate with tumor aggressiveness [147, 148]. ADC values correlate with mesorectal fascia invasion, lymph node involvement, histological differentiation, CA 19-9 and Ki-67 levels, and AgNOR counts [148, 149]. It also helps to differentiate between mucinous carcinoma and tubular adenocarcinoma [150].

DW-MRI is now more commonly used to assess early tumor changes and response after treatment [151, 152]. Treatment-induced cellular death and vascular changes can precede tumor size variation; thus, ADC variations may be a useful biomarker of treatment outcome for drugs that induce apoptosis and neoadjuvant CRT in locally advanced cancer [151]. In the literature, although controversial, it has been found that rectal cancer with low ADC values (<1.0 × 10–3 mm2/s) has a better response to CRT [153, 154]. Similarly, it has been demonstrated that liver metastasis with a high ADC baseline value shows a poor response to chemotherapy because it is commonly characterized by necrosis and cellular membrane disruption, suggesting an aggressive phenotype [155]. Another approach by Cai et al. showed that the signal intensity and signal intensity ratio of the tumor on DWI-MRI was more accurate than ADC measurements to assess complete tumor response [156].

Treatment-induced change is often preceded by perfusion alterations as changes of permeability, blood volume, and blood flow [157]. Because capillary perfusion influences the delivery of drugs to cancer cells, measurement of capillary perfusion by DCE-MRI is described as a surrogate marker for evaluating the efficacy of chemotherapy with bevacizumab [158]. Thanks to the fast imaging acquisition after intravenous contrast medium administration, DCE-MRI is an attractive modality for assessing antiangiogenic cancer treatments because it reveals changes in cancer vascularization and even predicts cancer shrinkage, otherwise reflecting a prognostic tumor phenotype [133, 158]. The improvement of postprocessing and the implementation of more complex algorithms of extraction of the signal decrease in DWI-MRI allow to separate tissue diffusivity and microcapillary perfusion. In detail, the biexponential model is based on the intravoxel incoherent motion (IVIM) theory introduced by Le Bihan et al., as a method useful to assess both perfusion and diffusion [159, 160]. This method could allow an early diagnosis of tumor response to CRT or new therapeutic agents like antiangiogenics [161, 162].

Heterogeneity is a well-recognized feature of malignancy associated with increased tumor aggression and treatment resistance. Texture analysis (TA) is an emerging image processing algorithm that can quantify heterogeneity of cancers. Recently, it has been reported that textural features of rectal cancer, assessed by textural analysis (TexRAD) using a filtration-histogram technique of T2-weighted pre- and post-CRT, can predict the outcome before undergoing surgery and could potentially select patients for individual therapy [163].

Malignant cells have high glucose metabolism, and the differential uptake of 18FDG by cancer cells can be used to detect both the short- and the long-term tumor responses, which either are not evident on CT or foresee a decrease in tumor size [164]. Integrated FDG-PET-CT provides complementary metabolic information that allows the detection of malignant disease in morphologically normal organs or at unexpected sites that can be easily overlooked on cross-sectional imaging [165]. The combination of metabolic and anatomical imaging increases sensitivity and specificity of cancer detection and is useful to evaluate treatment response [136, 166]. When assessed by FDG-PET-CT, metabolic response to therapy correlates with clinical response, tumor biology, and disease-free survival in CRC patients [166]. However, unlike CT scans, a validated scheme for assessing cancer response to therapy with FDG-PET-CT is not available [128]. In addition to FDG, other PET radiotracers can be employed to image intracellular processes targeted for therapy. Indicators of cellular proliferation include 18F-FLT, 11C-choline, and 18F-choline, whereas 15O-water and 18-F-FMISO indicate perfusion and hypoxia, respectively [136]. Hypoxia is known to contribute to CRT resistance, leading to angiogenesis and potential development of metastasis [165]. An imaging biomarker for radioresistance, such as 18-F-FMISO, could be employed to determine any differentials within the cancer and used to modulate radiotherapy in order to appropriately vary the radiation field and also to identify resistant areas that can be selectively dose escalated [137].

A novel approach of molecular imaging using PET-CT is the employment of radiolabelled antibodies or antibody fragments, such as 89Zr-rituximab, which allows to assess the distribution and availability among cancer cells of the epidermal growth factor receptor [167, 168]. This may support decision making for the selection of patients likely to benefit from therapy, identification of dose-limiting tissue, and optimization therapeutic planning [168].

Recently, thanks to new fusion technologies, a hybrid PET-MRI machine became available allowing functional imaging with simultaneously acquired PET and MRI data, changing the management of cancer patients [169–172]. This hybrid tool exceeds some limitations of FDG-PET-CT by allowing better soft tissue evaluation, more accurate T-staging, and improved characterization of small liver lesions and by providing better anatomical details for surgical planning while minimizing radiation exposure [169]. By adding functional MRI to PET, PET/MRI may further improve diagnostic accuracy in the differentiation of scar tissue for recurrence of CRC [172].

## 4. Nanotechnology and CRC Diagnosis

In recent years, nanotechnologies have made striking improvements in the diagnosis and treatment of human cancers. Specifically, they have enabled the development of nanomedicine, a new branch of medicine based on the use of nanomaterials in different activities, research, and clinical settings to improve the diagnosis and treatment of diseases [173]. Their applications in medicine are possible because nanoparticles (NPs) are resistant to oxidation, are easy to generate, and are full of interesting optical properties [174]. Additionally, other important characteristics such as biocompatibility, adaptable toxicity, dimension and surface chemical features, and a good stability in biological fluids and tissues [169] permit us to use them as active nanosystems in biomedicine [175–178].

In addition, because they are very small (nanometric scale), they are able to directly interact with cell and subcellular structures, although in a nonselective manner [175–177]. In principle, this could limit the utilization of NPs for specific applications; however, the organic groups and molecules linked to the NP surface (functionalization) allow to overcome this problem [174], thus improving the quality of the NP (based on the chemical groups linked on its surface, it can be used in different applications [174]). Further improvements to NPs include linking polyethylene glycol (PEG) molecules to its surface (to avoid passive extravasation and to increase their half-life in the circulation [168–180]) and the optimization of functionalization protocols that otherwise tend to generate agglomeration and agglutination of NPs [181, 182]. Finally, the addition of specific fragments capable of recognizing particular cell surfaces allows the NPs to transfer, accumulate, and promote the internalization of NP in a specific manner by tumor cells [183, 184].

### 4.1. Nanotechnologies in Noninvasive Diagnosis

Nanobioconjugates produced as previously described have been successfully used in the diagnosis of colorectal cancer [185]; in particular, various types of applications lead to an effective improvement of diagnostic techniques.

First of all, we know that, although MRI represents an indispensable noninvasive tool for diagnosis (it does not use ionizing radiation like computer tomography), it is still not sufficiently adequate to achieve 3D resolutions in real time [174]. However, this method is undergoing several improvements thanks to the use of magnetic nanobioconjugates as a contrast medium (magnetic particle imaging (MPI)), which help to increase the specificity and sensitivity of detection [174]. Among the NPs used effectively in these imaging techniques, we include iron oxide and nanobioconjugates constituted by liposomes, micelles, and dendrimers carrying paramagnetic ions [183, 186]. In addition, NPs have been used in a new noninvasive imaging technique (not employing ionizing radiation) defined as photoacoustic tomography, which combines ultrasound with the optical contrast provided by nanocages, carbon nanotubes, and gold speckled silica particles [187, 188]. Magnetic nanocrystals have also been used for multimodal diagnosis employing MPI and fluorescence [175]. As far as CRC is concerned, successful applications have been made with quantum dots (QD) [185]. These NPs are constituted by nanocrystals of a semiconductor and are capable of emitting fluorescence following excitation. Their particular optical characteristics provide them a series of advantages in several applications for cancer, including CRC [189, 190]. In particular, one study [191] reported an improvement of the immunohistochemical evaluation using QD in the procedure (QD-IHC). This methodology could be also applied to the immunocytochemical evaluation of antigens on the surface of living cells in a noninvasive manner, opening new perspectives in the evaluation of CTC in clinical practice [191]. Other studies demonstrated their eminent role in *in vivo* MRI diagnosis [192] and in CRC targeting of QD linked to bevacizumab [193]. In the latter case, noninvasive nanoprobes were able to detect CRC expressing high levels of VEGF. Additional methodologies, which take advantage of the useful characteristics of QD, are currently being evaluated and applied in the field of CRC diagnosis.

Another class of NPs employed in the analysis of CRC is constituted by dendrimers [185]. These are macromolecular structures that, starting from a centre, are formed by the addition of several repeated and branching elements. Similar to other NPs, these features conferred them the opportunity to be used in several applications of cancer nanomedicine [194, 195].

## 5. Bespoke Treatment: Role of Biomarkers in Clinical Setting

For many years, chemotherapy for mCRC was based simply on the combination of 5-fluorouracil (5-FU) plus levamisole (LV), a treatment that could improve median survival up to 11 months [196]. During the last 20 years, the association of oxaliplatin or irinotecan with 5-FU/LV led to an improvement in the outcome of patients affected by mCRC [197, 198]. Independently from the first-line therapy choice, patients receiving all available anti-CRC drugs may report a prolonged overall survival (OS) exceeding even 2 years [199]. The introduction of target therapies (bevacizumab, cetuximab, panitumumab, aflibercept, and regorafenib) has further ameliorated overall survival in the metastatic setting. As these agents are active on processes controlling cell growth, survival, angiogenesis, and spread following selective pathways, the efficacy of these drugs depends on a strict selection linked to particular molecular profiles.

The combination of mAbs binding to the vascular endothelial growth factor and EGFR with chemotherapy in mCRC has been shown to improve the efficacy, thus increasing treatment options [200, 201].

With the aim to optimize treatments, it is now well recognized that the variable responses among mCRC patients are influenced by the molecular profile of the tumor, which is specific and different among all individuals. Therefore, it is essential to individualize these different molecular aspects.

In the management of mCRC, several prognostic and predictive biomarkers have been identified over the past years and they can be used to define a personalised treatment for patients. Prognostic biomarkers identify patients regardless of treatment and may provide details about the disease prognosis. Predictive biomarkers help categorize patients potentially benefiting from a specific treatment or that show resistance [202] towards it. Thus, many analyses were conducted to identify tumor-related predictive factors aimed to suggest treatment responses [203].

The Erb family of cell membrane receptors includes HER1/erbB1 (EGFR), HER2/c-neu (ErbB-2), HER3 (ErbB-3), and HER4 (ErbB-4) [204]. As the EGFR gene was initially identified as an oncogene, it has become progressively the main target of biologic agents, prompting the development of anti-EGFR mAbs and tyrosine kinase inhibitors (TKIs). mAbs cetuximab (an anti-IgG1) and panitumumab (an anti-IgG2) act by binding to the extracellular domain site of the receptor, whereas erlotinib and gefitinib, two EGFR TKIs, compete with the binding site of ATP to the TK portion of the receptor, resulting in the inhibition of EGFR autophosphorylation. Both strategies (mAb and TKI) suspend the intracellular downstream signalling transmission.

The first clinical trials exploring the efficacy of anti-EGFR mAbs enrolled patients whose tumors expressed high levels of EGFR; however, overall response rates (ORRs) were low [205], which suggested the need of identifying additional factors potentially affecting the response to these agents [206]. Lièvre et al. were the first to identify a relation between mutant KRAS and poor responsiveness to EGFR-targeted treatments [207]. Thirty patients treated with cetuximab combined with irinotecan as second/third-line treatment were considered. KRAS mutations were detected in 13 of the 30 (43%) patients. None of the responders (0/11) had KRAS mutations, whereas 68.4% (13/19) of nonresponders presented them (*p* = 0.0003). The OS was significantly higher in wild-type KRAS (KRAS-WT) patients than in patients carrying a KRAS mutation (median OS: 16.3 versus 6.9 months, respectively, *p* = 0.016).

The next challenge was to understand why KRAS-mutated tumors did not respond to anti-EGFR mAbs. In this context, studies focused on key signalling molecule downstream of EGFR, including mutations in the KRAS, NRAS, BRAF, and PIK3CA genes and PTEN protein expression.

In the EGFR/RAS/RAF/MEK/ERK kinase downstream path, the KRAS protein is a GTPase that normally binds to the interior fragment of the cell wall. It conveys external signals from the receptor to the nucleus, regulating cell cycle (growth, proliferation, and apoptosis). The KRAS gene is located on the short arm of chromosome 12. Patients harboring point mutations in the KRAS gene generally have mutations within codon 12 at exon 2 (82%–87%), codon 13 (13%–18%), codon 61 (exon 3), and codon 146 (exon 4) [208]. In wild-type subjects, the activity of anti-EGFR mAbs on the external part of the receptor causes conformational changes blocking the RAS/RAF/MEK/ERK transmission. KRAS mutations impede EGFR activity for the constitutive activation of the intracellular fragment of the KRAS protein. In mCRC patients, the incidence of KRAS mutations is about 30%–45% [209].

Two randomized clinical trials comparing panitumumab or cetuximab with no active care in pretreated and chemorefractory mCRC patients [121, 208] demonstrated that KRAS mutant patients do not benefit from anti-EGFR mAbs. In the CO.17 [121] and AMGEN [208] studies, only KRAS-WT patients treated with cetuximab (median OS: 9.5 months versus 4.8 months) or panitumumab (median PFS: 12.3 weeks versus 7.3 weeks) had a survival benefit over best supportive care. In the cohort of patients with KRAS mutations, mAbs did not prolong PFS or OS.

The Cetuximab Combined With Irinotecan in First-Line Therapy for Metastatic Colorectal Cancer (CRYSTAL) phase III trial enrolled 599 patients to receive FOLFIRI plus cetuximab and 599 patients in the arm with FOLFIRI alone [210]. Sixty-four percent of the cases were exon 2-KRAS-WT; in these patients, both the risk of disease progression (HR of PFS: 0.68 [95% CI 0.50–0.94]) and that of death (HR of OS: 0.84 [95% CI 0.64 to 1.11] were lower in cetuximab-treated patients. No difference in PFS or OS was reported in the experimental arm in mutated patients.

The role of KRAS as a prognostic biomarker in CRC is quite controversial. The CO.17 study [121] analysed the prognostic involvement of KRAS status by assessing the interaction between KRAS status and survival in patients receiving best supportive care alone. There were no significant differences in median OS in either KRAS-WT or KRAS-MUT patients (4.8 months versus 4.6 months, resp.).

Similarly, Kim et al. [211] found that clinical outcomes did not differ between KRAS-WT and KRAS-MUT mCRC patients treated with chemotherapy alone. The RASCAL Collaborative Group evaluated the prognostic role of KRAS among thousands of patients with any-stage CRC [212, 213]. They found that KRAS-MUT patients presented shorter PFS and OS compared to wild-type patients. The RASCAL-2 study concluded that the G12 V mutation in the KRAS gene at codon 12 increases the risk of relapse or death only in Dukes' C CRC [213].

The retrospective analysis performed on mCRC patients in the MRC FOCUS trial [214] showed that KRAS mutations have a modest negative prognostic impact on OS (HR = 1.24; 95% CI 1.06–1.46; *p* = 0.008), but not on PFS (HR = 1.14; 95% CI 0.98–1.36; *p* = 0.09).

Neuroblastoma-ras (NRAS) is a member of the RAS oncogene family and is located on chromosome 1. The product of this gene is a GTPase enzyme membrane protein that shuttles between the Golgi apparatus and the cellular membrane. KRAS, BRAF, and NRAS mutations are mutually exclusive [215]. In CRC, the NRAS mutation rate is 3%–5% [216]. NRAS mutations are associated with the lack of response to cetuximab treatment. In the study by De Roock et al., NRAS-MUT patients treated with either cetuximab or panitumumab (2.6% of 644 KRAS-WT subjects) had a significantly lower ORR than NRAS-WT patients (7.7% versus 38.1%). PFS and OS did not differ statistically between mutated and wild-type patients.

A retrospective evaluation of biomarkers from patients enrolled in the PRIME trial indicated that NRAS plays an important role in predicting the efficacy of panitumumab treatment. Among the 656 patients with KRAS-WT exon 2, 108 (17%) had other mutations in KRAS exon 3 or 4, in NRAS exons 2, 3, or 4, or in BRAF exon 15 [217]. Patients with KRAS-WT exon 2 tumors bearing any RAS mutation did not achieve any benefit from panitumumab (median OS 17.1 months versus 17.1 months; *p* = 0.12). “All RAS” wild-type tumors (namely, wild type for KRAS exons 2/3/4 and for NRAS exons 2/3/4) significantly benefited from the combination treatment (median OS 25.8 months versus 20.2 months HR = 0.77; 95% CI 0.64–0.94; *p* = 0.009).

To elucidate the mechanisms of resistance to anti-EGFR antibodies, Bertotti et al. [218] performed a whole-exome analysis of 129 tumors in patient-derived xenografts detecting mutations in ERBB2, EGFR, FGFR1, PDGFRA, and MAP2K1 that could be potential mechanisms of primary resistance to anti-EGFR antibody therapy in CRC. Furthermore, investigation showed that amplifications and mutations in the tyrosine kinase receptor adaptor gene (IRS2) may contribute to the increased sensitivity to anti-EGFR therapy, representing a potential biomarker to predict full response to anti-EGFR-related CRC therapy.

Following these results, retrospective subanalyses were done on all the surviving wild-type patients enrolled in the CRYSTAL and OPUS trials. The results confirmed the important role of RAS mutational status in the optimal management of mCRC. Mutated patients do not benefit from anti-EGFR treatment, to the extent that this treatment could even be detrimental compared with chemotherapy alone. Therefore, in a real-life setting, the RAS mutational analysis has become essential and mandatory before beginning an anti-EGFR-based treatment.

### 5.1. BRAF

The BRAF protein is a cytoplasmic serine-threonine kinase bearing mutations in approximately 8%–10% of sporadic CRC [219]. The BRAF protein is one of the main effectors of KRAS; it is located immediately after KRAS effectors and it must be phosphorylated by KRAS to be activated. The point mutation V600E causes a CTG to CAG substitution at codon 600, which leads to a constitutive activation of the RAS/RAF/MEK/ERK cascade, similar to KRAS mutations.

In the retrospective analysis performed by Di Nicolantonio and colleagues, 113 tumor samples treated with cetuximab or panitumumab (with or without chemotherapy) were analysed and 79 KRAS-WT patients were identified. In this cohort, 11 (13.9%) patients were BRAF mutants and none of them responded to treatment.

In the CRYSTAL study [210], 9% (59 of 625) of patients carried BRAF mutations and they reported limited benefits from treatment with a shorter median OS in both arms, compared with the KRAS-WT and BRAF-WT population whose survival was 21.6 and 25.1 months, respectively. BRAF-MUT status was unrelated to cetuximab efficacy; thus, the authors concluded that BRAF mutation is a negative prognostic biomarker and not a predictive factor. Moreover, in the combined analysis of the CRYSTAL and OPUS results [220], a BRAF mutation was considered a negative prognostic marker in conclusion. In fact, survival times were lower in the BRAF-MUT population irrespective of therapy administered.

A BRAF mutation was also considered a negative prognostic biomarker in the PRIME trial [217]. In fact, patients with RAS-WT but BRAF-MUT tumors had a worse PFS and OS compared to subjects with wild-type RAS and BRAF tumors. In the RAS-WT/BRAF-MUT subgroup, the addition of panitumumab to chemotherapy produced a small benefit (difference was not statistically significant) in term of DFS and OS (*p* = 0.12 and 0.76, resp.).

The negative prognostic role of BRAF was also explored in clinical trials that enrolled patients to receive an intensive chemotherapy regimen plus an anti-VEGF treatment. The TRIBE trial compared the effect of bevacizumab plus the standard chemotherapy FOLFIRI with the association of 5-fluorouracil, irinotecan, levamisole, and oxaliplatin (FOLFOXIRI schedule). Final results of this trial showed a better outcome with the experimental arm; results showed an improvement of median PFS, OS, and ORR. A subset analysis was also performed in BRAF-MUT patients. Among the assessed patients, 28 (7%) BRAF-MUT patients reported a median OS of 13.7 months, significantly short if compared with the 37.1 months calculated for all wild-type patients [221].

It has been recently debated if a BRAF mutation could be considered a negative predictive factor too. Two meta-analyses were published in 2015 highlighting BRAF function. Pietrantonio et al. [222] assessed the negative predictive role covered by this mutation, which was mainly exerted towards anti-EGFR treatment. Rowland et al. [223] concluded that a BRAF mutation could only be considered as a negative prognostic biomarker (based on a not-significant interaction test and on the absence of a sufficient amount of data).

In conclusion, a BRAF mutation should be considered a negative prognostic biomarker rather than a negative predictive factor influencing anti-EGFR mAbs. BRAF-MUT patients have a poorer prognosis than BRAF-WT patients, irrespective of schedule of chemotherapy. These patients may benefit from anti-EGFR mAb treatment, but to a significantly lesser extent than BRAF-WT patients.

### 5.2. HER-2

The HER family of tyrosine kinase receptors consists of EGFR, HER2 (ErbB2), HER3, and HER4. They are responsible for cell survival and proliferation via signalling through the RAS-RAF-ERK and PI3K-PTEN-AKT pathways [224].

HER2 is a potential therapeutic target in patients with CRCs, and it is overexpressed in 25–35% of human breast cancers [225]. The level and incidence of HER2 overexpression in primary CRCs appear to be different.

In 2012, The Cancer Genome Atlas (TCGA) Network published the most comprehensive systematic molecular characterization of CRC to date, revealing genomic amplifications or mutations of the tyrosine kinase-encoding gene *ERBB2* in 7% of colorectal tumors, suggesting a novel potential therapeutic target for this cancer [226].

Several studies have assessed HER2 overexpression in CRC, with some reporting membranous expression, varying in the range 2.1–11% in [227–233], and others reporting cytoplasmic overexpression in the range 47.4–68.5% [230, 234, 235].

Kavuri and colleagues [236] studied the effect of ERBB2-targeted therapy in *ERBB2*-mutated CRC demonstrating that engineered intestinal cell lines that host *ERBB2* mutations are highly sensitive to irreversible EGFR/ERBB2 tyrosine kinase inhibitors, neratinib and afatinib, with these inhibitors inducing effective inhibition of ERBB2 and its downstream pathways.

Furthermore, xenografts from these cells lines were also sensitive to both neratinib and the combination of neratinib and trastuzumab. Interestingly, single-agent neratinib in a patient-derived xenograft (PDX) harboring *ERBB2* L866M mutation and amplification resulted in tumor stabilization and not in tumor regression as in the case of the combination of trastuzumab and neratinib. This result has been confirmed in another PDX harboring *ERBB2* S310Y mutation. Both PDX models were resistant to trastuzumab alone.

Anti-EGFR therapies, including cetuximab and panitumumab, have improved the prognosis of patients with CRC, particularly in the case of wild-type *KRAS* genes, in which these agents exhibit greater effectiveness [210, 237–239]. KRAS activities downstream the EGFR pathway and its spontaneous activation because mutation promotes cell proliferation despite the presence of anti-EGFR antibody [240].

In the previously described trial, sequencing CRC tumors with *ERBB2* mutation, Kavuri et al. [236] found that 50% (6/12) had a coccurring *KRAS* mutation.

Similarly, Kloth et al. [241] reported that three of 14 of *ERBB2*-mutated MSI CRC harbored *KRAS* mutation. Even though this cooccurrence could be justified in hypermutated MSI tumors, it is a surprising finding in the nonhypermutated tumors. Above all, several studies have exclusively evaluated mutant or amplified *ERBB2* as a target in tumors or models lacking such *KRAS* alterations. Further studies will be needed to better define both the etiology of this cooccurrence as well as the therapeutic consequences.

Based on promising preclinical studies in *ERBB2*-amplified CRC [242, 243], Siena et al. [244] conducted a phase II clinical trial of dual ERBB2 blockade. Patients with *ERBB2*-amplified, *KRAS* exon 2 wild-type, metastatic CRC who progressed after multiple lines of therapy, were treated with trastuzumab and lapatinib. Of 913 patients screened, 44 (4.8%) were found to be *ERBB2* amplified. Among 23 patients treated with dual anti-ERBB2 therapy, 8 (35%) patients had an objective response.

Similarly, trastuzumab and pertuzumab demonstrated response rates of 23% and disease control rates of 69% in the colorectal arm of a basket study [245]. These results were consistent with those of Valtorta et al [246].

Recent studies do suggest that HER2 overexpression by gene amplification may indeed be related to poor outcome in *KRAS* wt metastatic CRC patients treated with cetuximab or panitumumab [247].

In a study of 137 patient-derived xenograft (PDX) tumors, conducted by Hurwitz and colleagues, HER2 amplification was found in 13.6% of cases in patients with cetuximab-resistant, *KRAS* wild-type tumors [243].

Although patients with *HER2* amplification were resistant to anti-EGFR antibody therapy, other treatment strategies, including lapatinib or trastuzumab, can overcome cetuximab resistance in CRCs [242]. Finally, in addition to the previously reported trial conducted by Siena et al. [244], Deeken et al. [248] concluded that the combination of cetuximab and lapatinib provided a partial response in some patients with CRC who were resistant to anti-EGFR antibody therapy.

### 5.3. MSI-H

There are two molecular pathways in colorectal carcinogenesis. One is chromosomal instability (CIN) and the other is microsatellite instability (MSI) [249, 250].

High-level microsatellite instability (MSI-H) CRCs constitute approximately 15% of all CRCs in Western countries [251–253], more frequent in the early than the late stage of disease. The cause of MSI-H colorectal cancers is a deficiency of the DNA mismatch repair (MMR) system characterized by unstable microsatellites, a type of simple DNA sequence repeat. Its role consists in the postreplicative control of newly synthesised DNA strands and the correction of polymerase misincorporation events [254, 255].

MSI-H colorectal cancers can occur as sporadic tumors, because of methylation of promoter regions of the hMLH1 during tumorigenesis [256], or in the context of hereditary nonpolyposis colorectal cancer (HNPCC) or Lynch syndrome (LS) [257] with mutations of DNA MMR genes, primarily hMLH1, hMSH2, hMSH6, and hPMS2.

A defect in MMR is not manifested until both alleles of an MMR gene are inactivated (even if LS is dominantly inherited, a second hit on the other allele is required). MSI status can be determined by DNA testing. In particular, five microsatellite markers recommended by the National Cancer Institute (NCI) workshop have been used for MSI analysis: BAT25, BAT26, D2S123, D5S346, and D17S250 [256].

Two or more of the five markers are required to confirm the presence of MSI-H. Conversely, a low level of MSI (MSI-L) is assigned when only one unstable markers is detected. MSI-H CRC is known to have well-defined clinicopathological and molecular features. In fact, MSI-H CRC are preferentially located in the proximal colon and frequently associated with a less advanced cancer stage, extracellular mucin production, medullary carcinoma and poorly differentiated carcinoma, tumor-infiltrating lymphocytes, a Crohn's-like lymphoid reaction, and a *BRAF* V600E mutation [258–260]. Furthermore, it is associated with favorable survival in comparison with MSS/MSI-L. Interestingly, it is associated with chemotherapy resistance (i.e., adjuvant 5-FU-based chemotherapy) [261–265] but patients with metastatic disease are good candidates for immune-targeted therapy such nivolumab or pembrolizumab [266–268]. Conversely, several studies supporting MSI-H as a predictive factor for improved response to irinotecan- or irinotecan-based chemotherapy in CRC patients have been reported [269, 270].

## 6. Conclusive Remarks

CRC is a complex biological process involving multiple steps and genes, including genetic and epigenetic [271] factors, germline and somatic mutations, and chromosomal aberrations [272].

The three most important pathways of CRC carcinogenesis are the EGFR signalling pathway, with the involvement of KRAS and BRAF, the DNA mismatch repair (MMR), and the fields of epigenetics such aberrant hypermethylation and microRNAs (miRNAs) expression.

Over the recent years, several biomarkers of CRC have been proposed and encouraging progress has been made in our understanding the behaviour of CRC at a molecular level. Even if further validation studies are needed, assessing the role of biomarkers in experimental models and in patients could open new perspectives concerning a patient-tailored approach. Moreover, they could increase CRC screening uptake, given their limited invasiveness.

## Figures and Tables

**Table 1:** 

Potential applications of LB in CRC
Early diagnosis
Assessment of molecular heterogeneity of overall disease
Identification of genetic alterations for targeted therapy
Evaluation of tumor response after preoperative treatments
Monitoring of minimal residual disease
Assessment of evolution of resistance in real time

**Table 2:** Imaging Biomarkers.

Modality	Parameters	Application
CT	Anatomical and functional imaging (DCET-CT)	Staging and treatment response
MRI	Anatomical and functional imaging (DWI, DCE-MRI, TA)	Diagnosis, local staging, prognostic evaluation, treatment response
PET-CI	Metabolic and anatomical imaging	Diagnosis, staging and treatment response
PET-MRI	Metabolic and anatomical imaging	Diagnosis, staging, prognostic evaluation, treatment response
